# A Study of the Functional Outcome of Supplementation of Hamstring Graft With Anterior Half of the Peroneus Longus Tendon in Arthroscopic Anterior Cruciate Ligament Reconstruction

**DOI:** 10.7759/cureus.30138

**Published:** 2022-10-10

**Authors:** Harpreet Singh, Kamal Kumar Agarwal, Sangam Tyagi, Purvesh Bhrambhatt, Ammar Rampurwala, Rishit Unjia, Neel Agarwal

**Affiliations:** 1 Department of Orthopaedics, Geetanjali Medical College and Hospital, Udaipur, IND

**Keywords:** peroneus longus tendon, anterior cruciate ligament, reconstruction, arthroscopic, hamstring graft, ahplt, anterior cruciate ligament (acl)

## Abstract

Introduction: The present study was done to analyze the functional outcome, donor site morbidities, and associated parameters when using the anterior half of the peroneus longus for augmentation of an inadequate hamstring graft when performing arthroscopic anterior cruciate ligament (ACL) reconstruction.

Methods: Thirty patients with complete ACL tears were operated on. In all these patients, the thickness or length of the hamstring graft taken was found to be inadequate (less than 8 mm and 7.5 cm, respectively). So, additionally, the anterior half of the peroneus longus tendon (AHPLT) graft was harvested to reach an optimum size of the final graft. Functional outcome was assessed using the International Knee Documentation Committee (IKDC) score at six weeks, three months, and six months. The Foot and Ankle Disability Index (FADI) score at six months was used to assess ankle stability.

Results: The mean age in our study was 28.8 years with male predominance (73.33%). The mean operative time was 86.4 minutes. The mean hamstring graft thickness in our study was 6.5 mm, which improved to 9.12 mm after augmentation. The mean graft length after AHPLT augmentation was 9.38 cm. The mean IKDC score at six months was 87.35. At the end of six months, FADI scores were found within normal limits (range: 135-136) in all the patients. No complications were noted in any of the patients.

Conclusion: The AHPLT graft was always found to be sufficient enough for augmenting the hamstring graft to reach an acceptable thickness and length. There was no graft site morbidity and the ankle functional levels remained the same as preoperative levels, making it an excellent choice for augmentation of inadequate hamstring grafts.

## Introduction

The anterior cruciate ligament (ACL) is the main restraint in the knee to prevent anterior translation of the tibia over the femur [1] and plays an essential role in balancing the rotational and valgus forces. Rupture of this ligament usually results from noncontact, rotational, or deceleration injuries. In dislocation of the knee, the ACL is also injured along with other ligaments [2]. Untreated or ACL-deficient knees have a higher incidence of meniscus and articular cartilage injuries, which can lead to early osteoarthritis [3,4].

The ultimate aim of reconstruction of the ligament is to re-establish the stability of the injured knee and facilitate an early return to day-to-day activities, including sports, and to prevent the onset of osteoarthritis as well as damage to the meniscus [5-7]. During the previous decade, arthroscopically assisted techniques have been accepted for reconstructing the ACL [8,9]. In 1954, the development of a successful arthroscope substantially increased the better and minimal invasive technique for ACL reconstruction [10]. Since 1982, ACL reconstruction has frequently been performed arthroscopically [11]. Arthroscopic reconstruction of the ligament has the benefit of being minimally invasive, precision in graft placement, and less disturbance of native normal tissues, thereby offering faster recovery and recuperation, shorter hospital stays, and a very low infection rate.

A good amount of literature is presently describing successful ACL reconstruction with the use of donor autografts (hamstring tendon (HT), patellar tendon, and quadriceps tendon) and allografts (Achilles, tibialis anterior, patellar tendon, and HT). Amongst them, bone-patellar tendon-bone (BPTB) autograft and HT are the most commonly used. Despite being highly successful, BPTB is associated with concerns about donor site morbidity such as knee pain (mostly anterior side), extension lack, and fracture of the patella [8,9]. Whereas HT autografts have shown fewer complications on the surgical site, higher maximum load to failure, higher tensile strength, minimally invasive for graft harvest, minimum knee pain, and minimum perioperative pain (less than 5%) [10,11].

Several studies have revealed that hamstring grafts with a diameter of less than 8 mm are more prone to failure than thicker grafts and have a higher incidence of revision surgery [12,13]. Spragg et al. conducted a cohort study in which they reported that the proportion of cases requiring revision surgery was found to be on the higher side in which the graft diameter was on the lesser side (between 7.0 mm and 7.5 mm) [12]. Thus, in our study, we considered HT grafts of less than 8 mm in thickness to be inadequate. The peroneus longus graft has been suggested in recent times as an autograft for reconstruction [13]. However, the peroneus longus tendon (PLT) plays an important role as a stabilizer of the foot and ankle and that raises concerns regarding the long-term effects of PLT graft harvesting on ankle function and gait mechanics.

Therefore, in this study, we have proposed harvesting only the anterior half of the peroneus longus tendon (AHPLT) to reinforce the HT graft where the graft diameter was less, and the remaining portion of the PLT was left for normal function. Zhao et al. (2012) conducted a study describing the biomechanical features of the AHPLT along with its safety, adequacy, and effectiveness. They concluded that it was a good autograft, considering its bearing force, safety, and risks [14]. In addition, this graft was easy to harvest with minimal complications at the donor site in short- and mid-term reports.

In this study, we have used the AHPLT to augment the hamstring graft in arthroscopic reconstruction of ACL in patients where the quadrupled HT graft was found to be inadequate in terms of length or diameter. As different patients have different symptoms, functions, and sports activities, patients differ in a variety of knee problems as some have adequate musculature and some do not have adequate musculature. We have studied the functional outcome of the procedure as well as donor site morbidity and any associated complications.

## Materials and methods

The study was conducted in the department of orthopedics at a tertiary care hospital in South Rajasthan from September 2020 to December 2021 after approval from the Institutional Research Review Committee, Geetanjali Medical College and Hospital (approval number: GMCH/IRRC/PG20/2020-21/5603(38)) and taking informed consent from the patients. All the patients with complete ACL tears and those 18 years of age or older were included in this study. A complete ACL tear was confirmed by clinical tests and MRI.

The patients were informed that intraoperatively, if the thickness of the hamstring graft was found to be inadequate, then an AHPLT graft would be harvested to supplement the hamstring graft and the patient would be included in the study [14]. Patients who were excluded from the study were those with revision ACL surgeries, ACL tears with any fractures around the knee, fresh bony ACL avulsion injuries, patients with associated neuro-muscular disorders, and other significant internal derangements of the knee, except meniscal tears. For confirmation, the Lachman test, anterior drawer test, and classic pivot shift test were done as a routine thorough physical examination of the knee along with detailed clinical history and systemic examination. All routine blood investigations were sent and an X-ray and MRI of the injured knee were performed.

**Figure 1:**
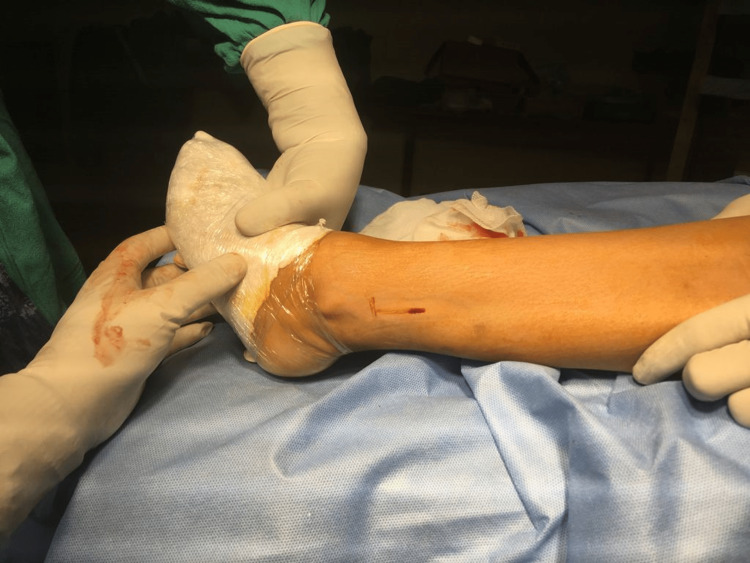
A 2-cm incision just proximal to the posterior border of lateral malleolus

**Figure 2:**
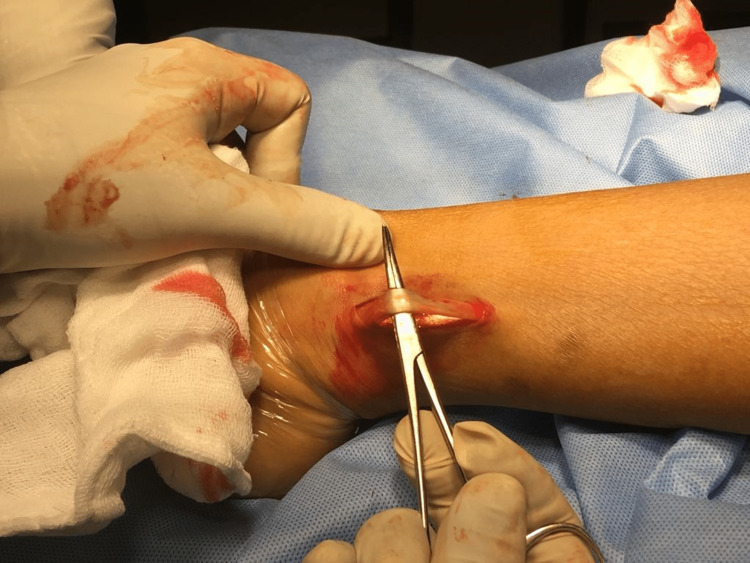
Isolating the peroneus longus tendon

**Figure 3:**
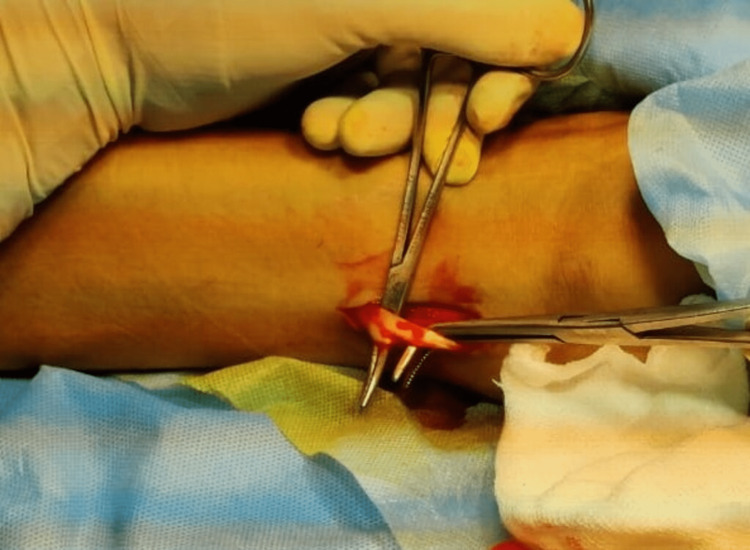
Anterior half of the peroneus longus tendon is split

Firstly, diagnostic arthroscopy was performed and any meniscal procedure required was done at this stage. Then the hamstring graft was harvested with a 3-cm oblique anteromedial incision on the tibia, the incision being taken approximately 3.5-4.5 cm distal to the knee joint line and 2.5-3.5 cm medial to the tibial tuberosity. Subcutaneous dissection was done, and after exposure of pes anserinus from its insertion, 2 cm medial to the tendinous insertion, gracilis and semitendinosus tendons were palpated and both the tendons were harvested after releasing their fibrous attachment. The ACL graft master was then used to prepare the grafts. In our study, we have taken grafts of a minimum size of 7 mm long and 8 mm in thickness. According to various studies, the graft should be at least greater than 8 mm for better functional outcomes [11,12]. If the above conditions were not met, we supplement the hamstring graft with an AHPLT graft. To harvest AHPLT, a 2-cm incision was made just proximal to the posterior border of the lateral malleolus with the foot in equinus and inversion over the prominent PLT (Figure 1). After splitting the fascia and isolating the peroneus longus (Figure 2), it was split into half and a thread was passed across its anterior half (Figure 3) [14,15]. We did not find any patients where we could not harvest AHPLT because of a smaller PLT.

**Figure 4:**
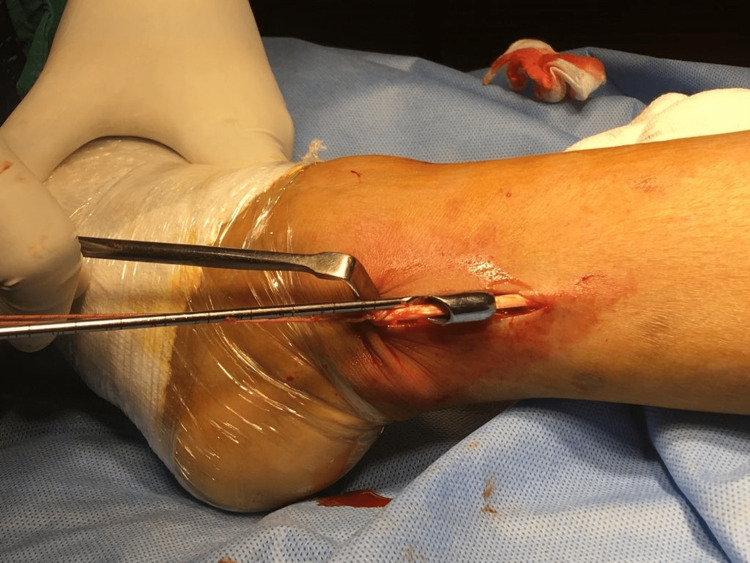
The anterior half of the peroneus longus tendon is harvested using a stripper

The distal end of AHPLT is then cut just above the lateral malleolus and it is then harvested using a stripper. The harvested AHPLT was then reinforced into the insufficient hamstring graft (Figure 4).

For the preparation of the femoral tunnel, with the arthroscope in the anterolateral portal, the femoral footprint of the ACL is identified in the medial wall of the lateral condyle, debrided, and marked. A guidewire is then drilled through the center of the anatomical footprint. The position of the guidewire was also checked with the arthroscope in the anteromedial portal. If found satisfactory, then drilling with a 4.5 mm cannulated drill bit was done "inside out." The length of the hole was then measured with a depth gauge and the size of the endo-button suspensory device was decided so that at least 2 cm of the graft remains in the femoral tunnel. The tunnel is then reamed with the reamer of the size of the thickness of the graft, at least 6 mm longer than the length of the graft to be retained inside the femoral tunnel, to allow the flipping of the endo-button. The tibial guide is then used to insert a guidewire from the medial part of the proximal tibia at a 25-degree angle from the midline in the coronal plane, at an inclination of 55 degrees in the sagittal plane, and made to exit on the lateral slope of the medial tibial spine. The tibial tunnel is then drilled to the same size as the thickness of the graft. The graft is then inserted through the tibial and femoral tunnels and fixed on the femoral side by an endo-button suspensory device and on the tibial side by a screw, after cycling the knee 20 times.

The entry for the tibial tunnel was made through the incision made for harvesting the hamstring graft. The tibial guide was positioned at the tibial footprint of the ACL located in line with the lateral slope of the medial aspect of the tibial spine, at the posterior aspect of the anterior horn of the lateral meniscus, and approximately 1.5 cm anterior to the PCL insertion at an angle of 50-60 degrees. The external part of the tibial guide was positioned flush with the anteromedial tibia, approximately 15 mm medial to the tibial tuberosity and 10 mm proximal to the pes anserinus attachment. After that, a 2.4 mm guide pin for the tibia was inserted through the guide until the tip of the pin was not visible protruding through the tibial footprint. Subsequently, the graft was passed through the tunnels thus made and was fixed on its femoral insertion site using an endo-button suspensory device and to that of the tibia by bio-interference and metal screws of suitable length and thickness. Wound closure was done in layers, a compression bandage was applied and a long knee brace was given.

Knee mobilization exercises were permitted from the evening after surgery and ambulation was allowed with a long knee brace. On postoperative day one, patients were put on the postoperative ACL rehabilitation protocol, which included knee mobilization exercises, quadriceps strengthening exercises, and ankle pumps. The goal was to achieve a 90-degree range of motion (ROM) in the first two weeks and a 120-degree ROM by the fourth week. The patients were followed up after two weeks for an inspection of the wound and the removal of sutures. The long knee brace was continued up to six weeks post-surgery and was advised to be worn only during ambulation.

The functional assessment of the patient was done using the International Knee Documentation Committee (IKDC) score and clinical examination for anteroposterior stability at six weeks, three months, and six months follow-up [16]. To clinically evaluate the ankle donor site of the AHPLT graft, the Foot and Ankle Disability Index (FADI) scores were used.

## Results

During the period of January 2020 to May 2021, 30 patients who were operated on for arthroscopic ACL reconstruction using hamstring graft required an AHPLT graft augmentation, and all these patients were studied. The age range of the patients was 18-46 years, with a mean age of 28.8 years. Half (50%) of these patients were in the age group of 21-30 years (Table 1).

**Table 1:** Demographic data (n =30) RTA: road traffic accident; PCL: posterior cruciate ligament.

Characteristics	Parameters	Data
Age distribution	<21 years	3
	21-30 years	15
	31-40 years	8
	41-50 years	4
Sex distribution	Male	22 (73.3%)
	Female	08 (26.6%)
Side of injury	Right	22 (73.3%)
	Left	08 (26.6%)
Nature of injury	RTA	11 (36.3%)
	Sports injury	12 (40%)
	Fall	7 (23%)
Associated injuries on MRI	Medial meniscus	14 (46.6%)
	Lateral meniscus	03 (10%)
	PCL	01 (3.3%)

In our series of 30 patients, male predominance was seen (73.33%). It may be because male patients are more active in sports and have higher incidents of road traffic accidents. In our study, 22 patients had right knee injuries, as most of the patients were right-sided dominant (73.2%) (Table 1).

The major source of injury was seen to be sports or physical activities (40% of the patients), followed by road traffic accidents (36.63%). Few patients (23%) sustained ACL injuries as a result of minor trauma during routine daily activities (Table 1).

All patients presented with the complaint of "giving way" of the knee. Other symptoms included pain (63%), swelling (53.3%), and locking of the knee joint (50%) (Table 2).

**Table 2:** Data of various parameters studied IKDC: International Knee Documentation Committee; FADI: Foot and Ankle Disability Index; RTA: road traffic accident; ACL: anterior cruciate ligament; PCL: posterior cruciate ligament; M: male; F: female; R: right; L: left.

Sr. no.	Age	Sex	Side	Mechanism of injury	Pain	Swelling	Locking	Giving away	MRI finding	Duration of surgery (min)	Final graft diameter (mm)	Final graft length (cm)	IKDC -preoperative	IKDC at 6 months	Active ankle ROM	FADI at 6 months
1	18	M	R	RTA	Yes	No	No	Yes	ACL	88	7 + 2	9	55.2	79	N	135
2	27	M	R	Sports injury	No	Yes	Yes	Yes	ACL + lateral meniscus	95		9.5	62.1	95.4	N	136
3	21	F	R	Fall	Yes	Yes	No	Yes	ACL	85	6 + 3	8	36.8	88.5	N	136
4	26	M	R	Sports injury	No	No	Yes	Yes	ACL + medial meniscus	99	7 + 2	9	50.6	86.2	N	136
5	46	M	L	RTA	Yes	Yes	No	Yes	ACL + PCL	94	6 + 3	9	52.3	87.2	N	135
6	35	M	L	RTA	Yes	Yes	Yes	Yes	ACL + medial meniscus	91	7 + 2.5	8.5	29.8	84.5	N	136
7	24	M	L	RTA	Yes	Yes	Yes	Yes	ACL + lateral meniscus	101	7 + 2	10	52.3	92	N	136
8	21	F	R	Sports injury	No	Yes	Yes	Yes	ACL + medial meniscus	96	6 + 3	10	34.6	89.6	N	135
9	40	F	L	Fall	No	Yes	No	Yes	ACL + medial meniscus	97	7 + 2	10	46.8	93.6	N	136
10	24	M	L	Sports injury	Yes	No	No	Yes	ACL	99	7 + 2.5	9.5	36.8	84.3	N	136
11	18	M	R	Fall	Yes	No	No	Yes	ACL	80	6 + 3	9	50.6	92	N	136
12	38	F	R	RTA	Yes	No	Yes	Yes	ACL + medial meniscus	85	7 + 2	10	48.2	80.4	N	135
13	36	M	R	RTA	Yes	No	Yes	Yes	ACL + medial meniscus	80	7 + 2.5	9.5	46.2	88.5	N	136
14	25	M	R	RTA	Yes	Yes	No	Yes	ACL	82	6 + 3	9.5	37.9	93.1	N	136
15	30	F	L	Fall	Yes	No	Yes	Yes	ACL + medial meniscus	75	7 + 2	10	55.2	83.9	N	136
16	35	F	L	Sports injury	Yes	No	Yes	Yes	ACL + medial meniscus	80	6 + 3	9	50.6	78.6	N	136
17	28	F	R	Sports injury	Yes	Yes	No	Yes	ACL	85	7 + 3	8.5	48.2	79.3	N	135
18	45	M	L	Sports injury	Yes	Yes	No	Yes	ACL	90	7.5 + 2	11	46.8	84.6	N	136
19	27	M	R	Fall	Yes	Yes	No	Yes	ACL	90	6 + 3	9	49.4	90.8	N	136
20	24	M	R	Sports injury	Yes	Yes	Yes	Yes	ACL + medial meniscus	85	6 + 3	9.5	50.5	89.6	N	135
21	34	M	R	Sports injury	No	No	Yes	Yes	ACL + medial meniscus	80	6 + 3	9	36.8	84	N	136
22	21	M	R	Fall	No	No	No	Yes	ACL	90	7 + 2	10	48.2	93.6	N	136
23	18	M	R	RTA	Yes	Yes	Yes	Yes	ACL + lateral meniscus	80	6 + 3	10	50.6	87.2	N	136
24	25	M	R	Sports injury	Yes	No	Yes	Yes	ACL + medial meniscus	85	6 + 3.5	8.5	37.9	92	N	136
25	24	M	R	Fall	Yes	No	No	Yes	ACL	75	7 + 2.5	9.5	50.6	89.6	N	136
26	29	M	R	RTA	No	Yes	No	Yes	ACL	80	7 + 2	9.5	46.8	93	N	136
27	33	M	R	Sports injury	No	No	No	Yes	ACL + medial meniscus	85	6 + 3	8.5	34.4	86	N	136
28	32	M	R	RTA	No	No	No	Yes	ACL	80	7 + 2	10	38	84	N	136
29	40	M	R	Sports injury	No	Yes	Yes	Yes	ACL + medial meniscus	85	6 + 3	10	48	88	N	136
30	21	F	R	RTA	No	Yes	Yes	Yes	ACL + medial meniscus	75	6 + 3	9	46	82	N	136

The medial meniscal tear was the commonest associated injury (46.6%) detected by MRI, followed by lateral meniscus tear (10%), and grade 1 posterior cruciate ligament injury not requiring surgery (3.3%). The mean operative time was 86.4 minutes (range: 75-101 minutes). All demographic data are shown in Table 1.

The mean graft diameter (thickness) in our study before augmentation was 6.5 mm and after augmentation with an AHPLT graft was 9.12 mm (range: 8.5-10 mm). The mean graft length after AHPLT augmentation in our study was 9.38 cm (range: 8-11 cm) (Table 3).

**Table 3:** Graft parameters (n = 30)

Parameters	Mean (range)
Graft diameter	Before augmentation - 6.5 mm (6-7.5 mm)
	After augmentation - 9.12 mm (8.5-10 mm)
Graft length	9.37 cm (8-11 cm)

We included all the patients with a graft diameter of <8 mm, and hence they needed additional supplementation with AHPLT. On performing clinical examinations at follow-up at six weeks, three months, and six months, none of our patients had instability on physical examination, as checked by various tests such as the anterior drawers test, the Lachman's test, and the pivot shift test. The mean preoperative and postoperative IKDC subjective scores were 45.94 and 87.35, respectively. As shown in Table 4, on comparing the preoperative and postoperative IKDC scores (p < 0.05), it was observed that postoperative IKDC scores showed significant improvement. We assessed all the patients at six months follow-up for ankle stability with the FADI score, which was near normal with a range of 135-136 (mean: 135.8), without any major complications (Table 4).

**Table 4:** Comparison of pre- and postoperative results IKDC: International Knee Documentation Committee; FADI: Foot and Ankle Disability Index.

	Preoperative mean	Postoperative mean	P-value
IKDC subjective score (out of 100)	45.94 (35-55)	87.35 (81-95)	0.00001
FADI score (out of 136)	136	135.8 (135-136)	

## Discussion

The rise in ACL injuries can be attributed to an increase in road traffic accidents and increased involvement in sports and physical activities like dancing. Along with the rise in incidence, the reconstruction of the ligament has also seen an uptrend with all the new advancements in technique and training.

There have been concerns that the harvesting of peroneus longus may affect ankle function. Therefore, only the AHPLT was harvested for augmentation, and it was seen that the thickness of AHPLT was sufficient enough to augment the hamstring grafts. All augmented grafts had a resultant thickness of at least 8.5 mm and a minimum length of 8 cm. The preoperative and postoperative values of FADI scores of all the patients showed an insignificant difference on clinical examination at six months. This means that ankle function and stability are not affected by harvesting the AHPLT. At six months, the FADI score of all patients was normal to near normal (range: 135-136) [17].

If left untreated, ACL tears lead to knee instability and potentially debilitating long-term sequelae. The improved results and excellent prognosis, leading to functional outcomes comparable to normal or near normal knees and ensuring a high probability of return to preinjury levels of sports and physical activities, make arthroscopic reconstruction the gold standard for ACL injuries [18].

Graft choice in arthroscopic reconstruction has been a matter of debate. Among the various available grafts, such as BPTB graft, hamstring autograft, quadriceps tendon, various synthetic grafts, and allografts, hamstring autografts had the minimum donor site morbidity. Multiple studies indicate higher strength, stiffness, and cross-sectional area when using multiple-strand hamstring autograft for ACL reconstruction [19-21].

In Pinczewski et al.'s 10-year comparative study of ACL reconstruction with HT and patellar tendon autograft, they reported that HT autograft is superior to BPTB autograft in postoperative knee stability, Lysholm score, and radiographic osteoarthritic change [21]. Helito et al. described the use of a quadrupled semitendinosus tendon graft with a bone block harvested from the tibial end of the tendon. Their results showed that the subjective rating by the patient was better with the HT graft than with the patellar tendon graft. The IKDC score and anterior knee pain favor the semitendinosus tendon as the graft material of choice [20]. Therefore, the hamstring graft has become the choice of most surgeons for arthroscopic ACL reconstruction.

In a study done by Spragg et al., it was observed that adequate graft diameter is between 9 mm and 10 mm, which significantly reduces revision surgery. In their cohort, the number of revision surgeries decreases with every 0.5 mm increase in graft diameter, from 7 mm to 10 mm, and the requirement for revision surgery decreases by 0.86 times when treated with hamstring autograft with adequate graft size [12]. In our study, we considered a hamstring graft thickness of less than 8 mm and a length of less than 7 cm to be inadequate, and in these patients, we used an AHPLT graft for augmentation. The average graft diameter without the AHPLT graft in our study was 6.5 mm and after augmentation was 9.12 mm. The average graft length was 9.38 cm.

The mean preoperative IKDC score in our study was 45.94, which improved to 87.35 at the six-month follow-up. This improvement in IKDC scores was statistically significant (p < 0.05). The final scores ranged from fair to excellent at six months (fair: 6, good: 23, and excellent: 1). We compared the IKDC score with studies of Kumar et al. (2016) [22], Khan et al. (2010) [23], and Aparajit et al. (2016) [24], and the postoperative scores in their study were 89.38, 94.33, and 86.03 respectively. We did a clinical examination with anterior drawers, Lachman test, and pivot shift test and did not notice any anteroposterior instability in any follow-up.

Our study showed that meniscal injury was seen in 56.6% of patients. Amongst the patients with meniscal injuries, the medial meniscus (n = 14) was injured more frequently than the lateral meniscus (n = 3). Many other studies have also reported the same findings [25]. Among the patients in our study, all meniscal injuries did not need surgery and were treated conservatively. Patients with an isolated ACL injury and those associated with meniscal injury were compared, and the functional outcome was found to be similar. A study by Lewis et al. also reported that the presence of meniscal injury does not alter the functional outcome [25].

We also faced various limitations in our study as we did not include patients with post-traumatic ankle, congenital talipes equinovarus, poliomyelitis, and congenital vertical talus.

During their time of stay or throughout the follow-up period, no complications were noted in any of the patients. No patellofemoral pain was noted in any patients, which is a regular observation made in studies of ACL reconstruction using the patellar tendon graft [8,9]. There were no donor site morbidities for the hamstring as well as peroneus graft sites when compared to the healthy side. The ankles showed a normal range of motion and normal power even on postoperative day one, and normal functional abilities throughout the follow-up. There were no incidences of graft tears or revision surgery in the period of follow-up. Many studies have reported a decreased revision rate when the graft diameter and length are kept above 8 mm and 7 mm, respectively [12].

## Conclusions

In conclusion, we would like to affirm that the AHPLT graft is an excellent choice for the augmentation of insufficient hamstring grafts when performing arthroscopic ACL reconstruction. There is no graft site morbidity and the ankle functional levels remain the same as preoperative levels. Also, it was seen that the AHPLT graft augmentation was always found to be sufficient enough to enhance the hamstring graft to reach an acceptable size and length of the final graft. Thus, we recommend that the use of AHPLT graft for augmentation of hamstring graft in ACL reconstruction is an excellent choice, safe, and gives desirable functional outcomes.
